# Effects of gestational age and the mode of surgical abortion on postabortion hemorrhage and fever: evidence from population-based reproductive health survey in Georgia

**DOI:** 10.1186/s12905-017-0495-7

**Published:** 2017-12-28

**Authors:** Ekaterine Pestvenidze, Nino Lomia, Nino Berdzuli, Lia Umikashvili, Tamar Antelava, Babill Stray-Pedersen

**Affiliations:** 1 Institute of Clinical Medicine, Rikshospitalet, Division of Women and Children, University of Oslo, Sognsvannsveien 20, 0372 Oslo, Norway; 2 Michener Institute of Health Sciences Ultrasound post-degree Diploma Program, 220 St Patric str, Toronto, ON M5A 3A1 Canada; 3 Tbilisi State Medical University, 33 Vazha-Pshavela Avenue, 0186 Tbilisi, Georgia; 4 Division of Women and Children, Rikshospitalet, Oslo University Hospital, Sognsvannsveien 20, 0372 Oslo, Norway; 5 Present address: 10 Jvania str. Apt 34, 0179, Tbilisi, Georgia

**Keywords:** Postabortion hemorrhage, Fever, Gestational age, Dilatation and curettage, Vacuum aspiration

## Abstract

**Background:**

Every year around 50 million unintended pregnancies worldwide are terminated by induced abortion. Even in countries, where it is legalized and performed in a safe environment, abortion carries some risk of complications for women. Findings of researchers on the factors that influence the sequelae of abortion are controversial and inconsistent. This study evaluates the effects of gestational age and the method of surgical abortion (i.e., dilatation and curettage and vacuum aspiration) on the most common abortion complications: postabortion hemorrhage and fever.

**Methods:**

We performed a secondary analysis of the data from the population-based Georgian Reproductive Health Survey 2010. Information on 1974 surgical abortions performed >30 days prior to the survey interview were analyzed during the study. Logistic regression statistical analysis was applied to compare the abortion sequelae that followed vacuum aspiration and dilatation and curettage at different gestational ages (<10 weeks and ≥10 weeks). We examined two major early abortion-related complications: postabortion hemorrhage and febrile morbidity (fever ≥38 °C).

**Results:**

Postabortion hemorrhage was reported in 43 cases (1.9%), and febrile morbidity occurred in 44 cases (2%) among all of the surgical abortions. The abortions performed by dilatation and curettage were associated with an estimated fourfold increased risk of developing hemorrhage (OR 4.4, 95% CI 2.2–8.6) and a twofold increased risk of developing fever (OR 2.37, 95% CI 1.17–4.79) compared with the abortions that were performed via vacuum aspiration. The risk of postabortion hemorrhage (OR 1.9, 95% CI 0.8–4.4) or fever (OR 0.9, 95% CI 0.4–2.1) did not significantly differ at gestational age < 10 weeks and ≥10 weeks.

**Conclusion:**

Vacuum aspiration was associated with reduced risks of postabortion hemorrhage and fever compared to dilatation and curettage. Gestational age ≥ 10 weeks was not found to be a predictive factor of immediate postabortion complications: hemorrhage and fever.

## Background

Eighty million women worldwide have an unintended pregnancy annually [1] and around 50 million of them are terminated by induced abortion [2]. Alarmingly high rates (~11 million abortions annually) were documented in the Soviet Union [3]. Georgia as a former soviet republic showed highest abortion estimates reported for any country according to the first population-based Reproductive Health Survey (RHS) in 1999 [4] - 3.7 abortions per woman, which declined to 3.1 in 2005 [5], and to 1.6 in 2010 [6]. Despite tangible progress, abortion in Georgia still plays a leading role in fertility control, which explains generated interest toward abortion-related morbidity and the factors that affect it.

An abortion complication in countries in which the procedure is legalized is primarily associated with infection, hemorrhage, and uterine injury [7]. Although the rate of abortion-related morbidity has been reported to be on average 9–10% [8, 9], the range varies markedly across the world from <1% to 15% as does the severity of complications [10–12].

Many researchers have studied the factors that influence the sequelae of abortion, including gestational age [13, 14], method of abortion [13], patient age [13, 15], and use of prophylactic antibiotics [16]. Gestational age and the method of surgical abortion have been reported to be the most significant and interrelated factors that cumulatively affect abortion-related morbidity [13]. However, the findings of the studies available are controversial and inconsistent.

In the United States, where abortion is safe, the risk of complications has been documented to increase exponentially with increasing gestational age [17]. Similarly, in a worldwide review, second trimester abortions were associated with significantly greater complication rates compared with first-trimester abortions (0.5–3% vs. 1–8%) [3]. Contradictory were findings of Jacot and associates in a comparative study of the complication rates of first and second trimester abortions, which documented no difference in the morbidity rates according to gestational age [18].

Method of abortion is another important factor that may affect abortion-related complications. The choice of the method to use (medical abortion, vacuum aspiration or dilatation and curettage (D&C)) depends on the setting, where abortion is performed, availability of the equipment, skills of the service providers, gestation at which a woman presents for abortion.

Aspiration of uterus is a recommended standard method for first-trimester surgical termination of pregnancy [19, 20]. However, some Georgian health care providers still prioritize D&C as a preferred technique for surgical abortion [7].

Few studies are available which directly compare D&C and vacuum aspiration as methods for surgical abortion and their findings are controversial. Some investigators have demonstrated that lower complication rates are associated with vacuum aspiration compared with D&C [21]. However, these results have been disputed by other researchers [22] who failed to find the described pattern.

The controversy and inconsistency regarding the findings of abortion-related morbidity studies may be explained by the variability in the complication tracking processes and the definitions of sequelae across the studies. The studies that have been conducted to date are limited by short-term findings. Most of them relied on hospital data to trace the medical sequelae of abortion and therefore only examined the immediate post-operative period, typically the first 24 h. Because the patients were not followed up for longer period, complications that may have emerged later often have been omitted [23].

The results of the largest population-based reproductive health survey in Georgia were studied with the aim of evaluating the effects of gestational age and the method of surgical abortion (D&C and vacuum aspiration) on the most common abortion complications. The study analyzed information on postabortion fever and hemorrhage collected from women minimum 30 days after the abortion. By allowing enough time for development of postabortion complications, the study addressed the limitation of previous researches related to the short-term follow-up of abortion-related complications.

## Methods

The study performed secondary analysis of the data from the population-based Georgian RHS 2010 that was conducted by the National Center for Disease Control and Public Health (NCDC&PH) in collaboration with the Georgian Ministry of Labor, Health, and Social Affairs (MoLHSA) and the Centers for Disease Control and Prevention (CDC), Atlanta, Georgia, USA (6).

The original survey employed large, nationally representative, probability sample (13,363 households). Information was collected through face-to-face interviews with women of reproductive age (15–44 years old) who were living in Georgia at the time of the survey.

### Study participants

The study targets were derived from the surveyed women who underwent surgical abortions. For the purpose of the study, surgical abortion was defined as a procedure that was performed with the intent of terminating a pregnancy using D&C or vacuum aspiration. The study included all reported cases of surgical abortion and excluded medical abortion, miscarriages and ectopic pregnancies.

To minimize recall bias regarding abortion-related complications, only abortions performed within the five years (2005–2010) prior to the interview were analyzed. To allow enough time for developing postabortion hemorrhage and fever, surgical abortions performed more than 30 days before the interview were included in data analysis. Thus, the final sample used for the study yielded a total of 1974 surgical abortions.

### Types of interventions

The two types of surgical abortion methods, that is, vacuum aspiration and D&C, were compared. The abortion sequelae that occurred at different gestational ages, that is, <10 weeks and ≥10 weeks, were also compared with each other.

### Outcome measures

Two major early abortion-related complications were traced during this study:

- Hemorrhage (defined as severe blood loss requiring blood transfusion)
- Febrile morbidity (defined as fever ≥38 °C).

### Data analysis

The collected data were analyzed using SPSS version 21.0 (IBM, Amonk, NY, USA).

We applied logistic regression analysis to examine the associations of the mode of abortion and gestational age with abortion-related complications: postabortion hemorrhage and fever.

In the initial stage, a number of potential predisposing social and medical factors/confounders derived from previous studies were considered for inclusion in the model. These factors were the following: age, ethnicity, educational status, employment, residence, wealth, parity, and the number of previous induced abortions.

Univariate regression analyses were performed, and crude odds ratios (ORs) were calculated for each of these variables. Subsequently, binary logistic regression was applied using the backward selection technique. All variables that were considered to be potential affecting factors were entered into the model, and those with the smallest contributions were removed sequentially until all the remaining variables contributed to the model. Ultimately, only the variables that were significant at a *P*-value <0.05 were retained in the final model.

## Results

Of 1974 surgical abortions analyzed, 652 (33%) were performed by D&C and 1322 (67%) by vacuum aspiration, 1687 (85%) at <10 weeks of gestation and 287 (15%) at ≥10 weeks of gestation.

Table 1 illustrates the socio-demographic factors and reproductive history of women performing surgical abortions according to method of abortion and gestational age.

**Table 1:** Selected characteristics of women with surgical abortions by method of abortion and gestational age

Characteristics	Method of abortion			Gestational age		
	D&C (*N* = 652) n (%)	Vacuum aspiration (*N* = 1322) n (%)	P	<10 weeks (*N* = 1687) n (%)	≥10 weeks (*N* = 287) n (%)	P
*Age (years)*						
15–24	73 (11.2)	137 (10.4)	0.173	174 (10.3)	36 (12.5)	0.237
25–34	359 (55.1)	786 (59.5)		991 (58.7)	154 (53.7)	
35–44	220 (33.7)	399 (30.2)		522 (30.9)	97 (33.8)	
*Residence*						
Urban	166 (25.5)	585 (44.3)	<0.001	681 (40.4)	70 (24.4)	<0.001
Rural	486 (74.5)	737 (55.7)		1006 (59.6)	217 (75.6)	
*Education level*						
Secondary incomplete	174 (26.7)	255 (19.3)	<0.001	341 (20.2)	88 (30.7)	<0.001
Secondary complete	225 (34.5)	408 (30.9)		544 (32.2)	89 (31.0)	
Professional college	111 (17.0)	170 (12.9)		235 (13.9)	46 (16.0)	
University	142 (21.8)	489 (37.0)		567 (33.6)	64 (22.3)	
*Wealth quintile*						
Lowest	200 (30.7)	211 (16.0)	<0.001	316 (18.7)	95 (33.1)	<0.001
Second	171 (26.2)	301 (22.8)		403 (23.9)	69 (24.0)	
Middle	159 (24.4)	325 (24.6)		417 (24.7)	67 (23.3)	
Fourth	41 (6.3)	229 (17.3)		249 (14.8)	21 (7.3)	
Highest	81 (12.4)	256 (19.4)		302 (17.9)	35 (12.2)	
*Ethnicity*						
Georgian	506 (77.6)	1105 (83.6)	<0.001	1398 (82.9)	213 (74.2)	<0.001
Azeri	71 (10.9)	89 (6.7)		131 (7.8)	29 (10.1)	
Armenian	56 (8.6)	77 (5.8)		102 (6.0)	31 (10.8)	
Other	19 (2.9)	51 (3.9)		56 (3.3)	14 (4.9)	
*Employment*						
Employed	98 (15.0)	252 (19.1)	0.027	298 (17.7)	52 (18.1)	0.852
Unemployed	554 (85.0)	1070 (80.9)		1389 (82.3)	235 (81.9)	
Other	0	0		0	0	
*Parity*						
0	3 (0.5)	4 (0.3)	<0.001	5 (0.3)	2 (0.7)	<0.001
1	89 (13.7)	226 (17.1)		261 (15.5)	54 (18.8)	
2	347 (53.2)	825 (62.4)		1032 (61.2)	140 (48.8)	
*3*	151 (23.2)	207 (15.7)		298 (17.7)	60 (20.9)	
4+	62 (9.5)	59 (4.5)		91 (5.4)	31 (10.8)	
*Number of previous induced abortions*						
1	206 (31.6)	384 (29.0)	<0.001	499 (29.6)	91 (31.7)	0.196
2	172 (26.4)	366 (27.7)		456 (27.0)	82 (28.6)	
3	119 (18.3)	221 (16.7)		285 (16.9)	55 (19.2)	
*4+*	155 (23.8)	351 (26.6)		447 (26.5)	59 (20.6)	

Highly educated women with higher incomes who were from urban areas tended to have abortions at a lower gestation age (<10 weeks) and through vacuum aspiration (*P* < 0.05). The differences were more marked in women with four or more complete pregnancies. These women were nearly twice as likely to undergo an abortion by D&C and at a gestational age ≥ 10 weeks than by vacuum aspiration and at a gestational age of <10 weeks (*P* < 0.05).

The total complication rate (from hemorrhage or fever) among surgical abortions by D&C or vacuum aspiration was 3.9% (77 cases).

The number of uterus perforations was too small (2 cases in total) to allow analysis of this complication according to method of abortion or gestational age.

### Hemorrhage

Postabortion hemorrhage was reported in 43 cases (1.9%). The rate was significantly higher after D&C (4.1%) than after vacuum aspiration (1.2%, *P* < 0.05). Abortions at gestational ages ≥10 weeks were not associated with an increased rate of hemorrhage compared with the abortions at <10 weeks of gestation (2.8% vs. 2.1%, respectively, *P* = 0.44, Table 2).

**Table 2:** Incidence of postabortion complications according to method of abortion and gestational age (*N* = 1974)

Postabortion complications	Method of abortion			Gestational age		
	D&C *n* = 652	Vacuum extract. *n* = 1322	P	<10 weeks *n* = 1687	≥10 weeks *n* = 287	P
Hemorrhage n (%)	27(4.1%)	16(1.2%)	<0.001	35(2.1%)	8(2.8%)	0.444
Fever n (%)	24(3.7%)	20(1.5%)	0.002	33(1.9%)	11(3.8%)	0.046

Table 3 illustrates the results of the regression analysis. The univariate analyses showed that the risk of postabortion hemorrhage was highest for the D&C procedure and for women with history of more than four abortions. Age, residence, education, wealth, ethnicity, employment and parity had no significant effect on postabortion hemorrhage (*P* > 0.05). Gestational age was also found to be a non-significant factor regarding hemorrhage after an abortion.

**Table 3:** Univariate and multivariate analyses of the potential risk factors for a postabortion hemorrhage

Risk factors	Univariate analysis				Multivariate analysis			
	Crude OR	CI 95%		P	Adjusted OR	CI 95%		P
		Lower	Upper			Lower	Upper	
Age	1.277	0.783	2.081	0.327				
Residence	0.964	0.516	1.802	0.909				
Education	0.988	0.863	1.131	0.861				
Wealth	0.931	0.747	1.160	0.525				
Ethnicity	1.024	0.814	1.289	0.838				
Employment	1.062	0.488	2.310	0.879				
Parity	0.972	0.651	1.450	0.888				
# of induced abortions	1.338	1.012	1.769	0.041	1.330	1.006	1.759	0.046
Method of abortion	3.526	1.886	6.592	<0.001	4.397	2.239	8.638	<0.001
Gestational age	0.739	0.339	1.609	0.446	1.909	0.823	4.428	0.132

After adjustments for the number of induced abortions, the binary logistic regression model revealed that the abortions performed by D&C were associated with an estimated fourfold increase in the risk of developing a hemorrhage (OR 4.4, 95% CI 2.2–8.6) compared with abortions that were performed via vacuum aspiration.

Even after the adjustment, gestational age did not significantly influence postabortion hemorrhage.

### Fever

Febrile morbidity (fever ≥38 °C) occurred in 44 cases (2%) among all of the surgical abortions performed via D&C and vacuum aspiration. The fever rate was found to be twice as high following D&C (3.7%) than following vacuum aspiration (1.5%, *P* < 0.05). A twofold increase in fever incidence was also observed following the abortions performed at or after the gestational age of 10 weeks compared with those performed before 10 weeks (3.8% vs. 1.9%, respectively, *P* = 0.046, Table 2).

The results of univariate regression analysis regarding the effects of various factors on postabortion fever are demonstrated in Table 4. The women with higher number of births, history of induced abortion, and D&C procedure were at the greatest risk of developing postabortion fever (*P* < 0.05). Age, residence, education, wealth, ethnicity and employment did not significantly affect the rate of fever (*P* > 0.05). The difference in fever between the abortions performed at <10 and ≥10 weeks of gestation was not significant (*P* > 0.05).

**Table 4:** Univariate and multivariate analyses of the potential risk factors for a postabortion fever

Risk factors	Univariate analysis				Multivariate analysis			
	Crude OR	CI 95%		P	Adjusted OR	CI 95%		P
		Lower	Upper			Lower	Upper	
Age	1.139	0.702	1.848	0.599				
Residence	1.026	0.555	1.895	0.935				
Education	0.917	0.797	1.055	0.225				
Wealth	0.844	0.679	1.048	0.125				
Ethnicity	1.079	0.841	1.384	0.550				
Employment	0.728	0.305	1.736	0.474				
Parity	1.596	1.037	2.458	0.034				
# of ind. Abortions	1.453	1.093	1.932	0.010	1.439	1.082	1.915	0.012
Method of abortion	2.488	1.364	4.538	0.003	2.370	1.171	4.795	0.016
Gestational age	0.501	0.250	1.002	0.051	0.934	0.414	2.112	0.871

Table 4 also presents the results of the final binary logistic regression analysis with the outcome variable of fever. After applying backward stepwise regression, the final model contained only two significantly contributing variables: the method of abortion and the number of previous induced abortions. Gestational age was included in the model, but had no significant influence. After adjusting for the number of induced abortions, the abortions performed by D&C were associated with a 2.37-fold increased risk (OR 2.37, 95% CI: 1.17–4.79) of developing fever compared with the abortions performed via vacuum aspiration.

Gestational age was not significantly associated with either postabortion hemorrhage or with postabortion fever.

## Discussion

The early sequelae of abortion, including uterine perforation, hemorrhage, and infection, and the factors affecting these sequelae have been studied by many researchers; however, the conclusions drawn from these studies are diverse and frequently controversial. The disparities in the findings might be explained by variability in the postabortion follow-up periods across the studies and ill-defined or disparate definitions of abortion-related complications.

The present study does not suffer from the limitation of a short follow-up period. The data regarding abortion complications were collected through direct interviews with the women who received the service minimum 30 days prior to the interview, which allowed sufficient time for the development of postabortion hemorrhage and fever.

The study compared the occurrence of hemorrhage and fever after abortions performed through vacuum aspiration versus D&C and before versus after the gestational age of 10 weeks.

### Method of abortion: Vacuum aspiration vs. D&C

In our study, D&C abortions were associated with increased risks of both postpartum hemorrhage and fever compared with vacuum aspiration, which contradicts with the results of meta-analysis of the trials conducted by Lean et al. [22] with 420 women and by Schweppe et al. [7] with 47 women. These studies found no significant differences in excessive blood loss or febrile morbidity following abortions performed with either vacuum aspiration or D&C. However, the interpretations of these studies should be approached with caution due to the small sample sizes and rarity of the outcomes, which likely hindered the detection of meaningful differences.

In a study of 4463 women, Edelman and associates found variability in the complication rates according to method of abortion at different gestational ages: prior to 12 weeks of pregnancy, vacuum aspiration was associated with reduced risks of excessive blood loss, prolonged bleeding and pelvic infection. After 12 weeks of gestation, the groups did not differ significantly in terms of complication rates [21].

### Gestational age: <10 weeks vs. ≥10 weeks

In the present study, gestational age was not found to strongly influence the development of postabortion complications. The risks of hemorrhage and fever did not significantly differ before and after 10 weeks of gestation.

These findings do not agree with previous data, indicating that complication rates invariably increase with increased gestation duration. The results of a study from Tietze et al. documented a dramatic growth of complication rates with higher gestation [24]. Moberg and associates also reported a notable rise in postabortion hemorrhage with increased gestational age among 1123 women who had undergone vacuum aspiration abortions. This increase was particularly pronounced after 11 weeks of gestation [25].

In a study by Ferris and colleagues [23] that examined post-abortion complications in Canadian (Ontario) abortion clinics via retrospective analyses of a provincial database of 83,469 abortions, the odds ratio for abortion complications was 1.3 (95% CI 1.02–1.63) between 9 and 12 weeks of gestation compared to the complications that occurred at <9 weeks and increased dramatically with further increase in gestational age. However, the findings of this study were based on a very short-term follow-up period (a few hours after the procedure), while even short-term postabortion complications may appear for up to several days after the procedure, and such complications were likely not represented in this study.

Our study results are supported by those of Jacot et al. (18), which did not find an increase in the risk of complications with increased gestational age. However, the research was limited to only a single clinic and to abortions that were performed under local anesthesia, which makes the findings not generalizable.

### Study limitations

The present study has several limitations. First, our findings relied solely on the responses of the women and were not verified through medical records. Hence, we do not know whether all of the respondents coded their complications similarly, although standard explanation of the complications was provided to each respondent by the interviewers. Second, because the information about abortion complication was collected from women who were interviewed months or even years after the abortions, the possibility of recall bias is present. To minimize the risk of recall bias, we only analyzed information about postabortion complications that occurred within five years prior to the interview.

## Conclusion

The absolute risk of complications after abortion is generally low when the termination of pregnancy is performed and managed by a skilled provider. The D&C procedure was found to be associated with increased risks of developing postabortion hemorrhage and fever compared with the vacuum aspiration procedure. Unlike the results of previous studies, increased gestational age (≥ 10 weeks) was not found to be a predictive factor of immediate postabortion complications: hemorrhage and fever.

There is a distinct need to define postabortion complications in a uniform manner and to establish uniform criteria for abortion follow-up period to make the study findings comparable and consistent.

## Abbreviations

CDC: Centers for Disease Control and Prevention
D&C: Dilatation and curettage
IRB: Institutional Review Board
MoLHSA: Ministry of Labor, Health, and Social Affairs
NCDC&PH: National Center for Disease Control and Public Health
RHS: Reproductive Health Survey
